# From RAMP to Triplex RT-qPCR: Modernizing Arbovirus Surveillance and Confirming the First *Aedes aegypti* in Idaho

**DOI:** 10.3390/pathogens15040406

**Published:** 2026-04-08

**Authors:** Heather M. Ward, James J. Lunders, Chris Ocegueda

**Affiliations:** Canyon County Mosquito Abatement District, Nampa, ID 83686, USA

**Keywords:** mosquito surveillance, arbovirus testing, West Nile virus, RT-qPCR, triplex PCR, molecular diagnostics, *Culex* spp., *Aedes aegypti*, mosquito abatement, surveillance capacity-building

## Abstract

West Nile virus (WNV) remains the most frequently reported locally acquired arboviral infection in the United States, yet many small and mid-sized mosquito abatement districts lack the diagnostic capacity and integrated data systems needed for rapid detection and response. The Canyon County Mosquito Abatement District (CCMAD) in southwestern Idaho undertook a multi-year capacity-building effort to expand arbovirus surveillance, standardize mosquito identification and pooling procedures, and implement triplex RT-qPCR testing for WNV, Western equine encephalitis virus (WEEV), and St. Louis encephalitis virus (SLEV). Historical trapping datasets (2021–2025) were consolidated, geospatially harmonized, and grouped into biologically meaningful sampling units to enable multi-year spatial comparisons. Surveillance revealed recurrent WNV activity annually, with peak transmission occurring between epidemiological weeks 31 and 37. The highest WNV activity occurred in 2023 and 2025, with 192 and 92 positive pools, respectively, while no WEEV or SLEV detections were observed. Enhanced laboratory capacity reduced sample-processing times, decreased the reliance on external confirmatory testing, lowered per-pool testing costs, and enabled same-day reporting to operational staff. In 2025, routine gravid trap surveillance detected a single *Aedes aegypti*, which was identified morphologically and subsequently confirmed by DNA barcoding, prompting targeted follow-up trapping. CCMAD’s integrated approach provides a scalable model for strengthening local surveillance and response capabilities in resource-limited settings.

## 1. Introduction

West Nile virus (WNV) has remained the most widely reported locally acquired arboviral infection in the United States for more than two decades, with sustained circulation in western states including Idaho [1]. Since its initial detection in Idaho in 2003 from 14 alligators [2,3,4], recurring seasonal transmission has affected human, equine, and mosquito populations. Historically, Idaho has seen evidence of other vector-borne pathogens as well. The first isolations of Western equine encephalitis virus (WEEV) occurred in the early 1970s [5]. However, these initial isolations of the virus were the result of a black bear survey, and were not from tested mosquitoes. St. Louis encephalitis virus (SLEV) was also detected in this survey, with the first report of SLEV in Idaho mosquitoes in 2017 [6]. Similarly, it would take 2 years after the first human case of WNV in the state before the 1st mosquito pool in Idaho tested positive for WNV [4].

Effective public health response depends on the ability of local mosquito abatement districts to detect viral activity early, characterize spatial and temporal trends, and deploy targeted vector control measures [1,7,8]. In fact, the California Department of Public Health concluded that the early detection and mitigation of WNV outbreaks within the state have been due to a strong partnership with local mosquito control agencies [9]. Furthermore, the Centers for Disease Control and Prevention (CDC) and the Environmental Protection Agency (EPA) both emphasize that local mosquito control organizations provide critical surveillance and control activities during emergencies or natural disasters [10]. Yet many districts, particularly small or mid-sized programs, face operational constraints including limited molecular diagnostic capacity, fragmented historical datasets, and inconsistent site metadata that complicate multi-year analysis [11,12]. As a result, arboviral pathogen presence in mosquitoes may go undetected and not provide a predictive measure for human cases.

The Canyon County Mosquito Abatement District (CCMAD), located in a rapidly developing region of southwestern Idaho, conducts integrated mosquito surveillance across diverse ecological interfaces ranging from irrigated agriculture to expanding urban corridors. As the County’s geography developed and changed with urbanization, CCMAD’s surveillance program necessarily adapted. However, without a unified direction through staff changes, site histories were lost and historical mosquito and viral trends were lacking. A key component of this modernization effort was an initial assessment of historical, disparate trap locations and naming conventions. Over the years, traps were added or moved, with new site names continually added, resulting in an exhaustive dataset of geographically similar, yet distinct records. Aggregating these geographically similar sites into biological site groups would allow for a multi-year comparison of consistent spatial footprints despite historical variation in site naming or minor relocations.

In addition, prior to 2023, CCMAD relied primarily on an immunochromatographic antigen-detection assay (e.g., RAMP^®^) and external services for RT-qPCR confirmation of WNV results, as well as testing for SLEV and WEEV. These approaches had higher per-test costs, extended processing and reporting times, and performance characteristics that limited scalability during peak transmission periods [6,13]. CCMAD, in line with other mosquito abatement programs nationwide, identified a need to increase our public health laboratory capacity with the addition of in-house RT-qPCR assay capabilities. These improvements would support local vector surveillance and diagnosis capabilities [1] to increase operational efficiency in response to detections of WNV, WEEV, and SLEV in local mosquito populations.

Critical to this overall modernization initiative was developing a dedicated staff with appropriate training in entomological identification and desired diagnostic testing capabilities. Properly trained and experienced staff would enable rapid and accurate species identification and pathogen detection that could directly inform specific control actions. Having the ability to rapidly distinguish vector from nuisance species, as well as detect invasive species, and confirm disease risk through improved testing platforms would support threshold-based integrated mosquito management actions. Over time, the resulting local dataset and institutional expertise should improve operational efficiency, reduce the costs associated with delayed or misdirected control efforts, and ensure that CCMAD can respond quickly to emerging and invasive vectors or outbreaks without relying on external laboratories.

In summary, beginning in 2022, CCMAD initiated a multi-year capacity-building effort that included:

1. Hiring dedicated staff with expertise in entomological surveillance and diagnostic testing platforms.
2. Standardizing mosquito identification, pooling procedures, and data management.
3. Validating a triplex RT-qPCR assay for the simultaneous detection of WNV, WEEV, and SLEV.
4. Expanding their laboratory facilities.
5. Establishing automated RNA extraction workflows.
6. Consolidating long-term trap and positive pool historical data into a unified geospatial dataset.

Here, we summarize CCMAD’s surveillance capacity improvements, current outcomes, and geospatial data analysis from 2021 to 2025 for multi-year spatial clustering of WNV activity using harmonized biological sites. Expanding the laboratory capacity allowed for an increased operational responsiveness and cost savings as in-house arbovirus testing capacity increased. With the expanded surveillance capacity, we have also documented the first confirmed detection of *Aedes aegypti* in Idaho, an invasive vector whose range has expanded across the United States in recent years [14,15]. This integrated approach for expanding the capacity for mosquito population and arbovirus surveillance provides a model for other local districts, with goals to strengthen their program effectiveness and public health responsiveness.

## 2. Materials and Methods

### 2.1. Mosquito Collection and Field Operations

Surveillance for adult mosquitoes relied on the weekly placement of encephalitis virus surveillance traps (EVS) (John W. Hock Company, Gainesville, FL, USA), Frommer updraft gravid traps (John W. Hock Company, Gainesville, FL, USA), and BG-Sentinel 2 traps (Biogents AG, Regensburg, Germany). BG-Sentinel 2 traps are deployed as needed according to district priorities for the collection of *Culex* mosquitoes and invasive *Aedes* monitoring. EVS traps and BG-Sentinel 2 traps were baited with approximately 3 pounds of dry ice. Gravid traps were baited with putrid water (approximately 140 g chicken feed and 15 g brewer’s yeast per 3.8 L of water). Traps were placed in the late afternoon according to manufacturer guidelines, in areas known to harbor mosquitoes, and were retrieved the following morning. Field staff recorded metadata including trap type, GPS coordinates, collection date, site name, and unique site code with each collection event (Appendix C). Because historical trap names and locations have varied across seasons and staff, raw coordinates were preserved and used for downstream spatial reconciliation (see Section 2.9 below).

### 2.2. Mosquito Handling and Identification

All trap collections were transported to the District’s identification laboratory on dry ice and stored in a standard chest freezer (PS-CFR102-I6A, Professional Series, China). For identification and enumeration, mosquitoes were euthanized by freezing and sorted by sex and species. Female mosquitoes were identified morphologically using national and regionally validated keys [16,17,18,19,20]. Identification focused on the District’s primary vector species (*Culex pipiens*, *Cx. tarsalis*, *Cx. erythrothorax*) and local pestiferous *Aedes*, *Anopheles*, and *Culiseta* species. Voucher specimens were pinned and retained on-site for quality assurance. In 2025, through standard morphological identification, a single *Ae. aegypti* was identified [18,19,21]. Because the specimen was badly damaged and it would represent the first detection of this species in Idaho, further DNA confirmation assays were performed to confirm the identification. The *Ae. aegypti* confirmation workflow is summarized in Section 3. 

### 2.3. Pooling Strategy

Female Culex were pooled by date, site, and species, following standard operational pool limits (≤50 individuals per vial). No mixed-species pools were included. Pool size limits and single-species pooling were selected to align with CDC recommended arbovirus surveillance standards intended to balance detection sensitivity, laboratory throughput, and operational feasibility [8]. Pools were stored at −80 °C in sterile 2.0 mL screw-cap homogenization tubes containing one copper-coated BB until homogenization. Metadata captured for each pool included species identification, a unique site ID, site name, trap type, collection date, and notes on the trap’s status upon collection (i.e., operational, operational but empty, malfunctions and tampering).

### 2.4. Mosquito Homogenization

Pools were homogenized in 800 µL viral transport medium using a Bertin Precellys 24 bead-mill homogenizer (Bertin Corp., Rockville, MD, USA). Homogenization parameters were optimized for mosquito tissues and performed in two 20 s cycles at 6000 rpm, with a 5 s pause between cycles. Homogenates were clarified through centrifugation prior to RNA extraction at 13,000 rpm for 3 min.

### 2.5. RNA Extraction

RNA extraction was performed using the QIAamp Viral RNA extraction mini kits (Qiagen, Germantown, MD, USA) following the manufacturer-defined centrifuge extraction protocol during 2023 through to June of 2025. A total of 140 µL of clarified homogenate was used, and RNA was eluted into 50 µL of elution buffer. In 2025, RNA extraction was also performed on a QIAvac 24 vacuum manifold (Qiagen, Germantown, MD, USA) system utilizing the QIAamp Viral RNA Extraction Mini kits with the same homogenate and elution volumes and followed the vacuum manifold extraction protocol. In late 2025, RNA was also extracted on a KingFisher Flex (ThermoFisher, Life Technologies Corporation, Carlsbad, CA, USA) automated nucleic acid system with the MagMAX CORE extraction kits (ThermoFisher, Life Technologies Corporation, Carlsbad, CA, USA) following a protocol provided by ThermoFisher’s technical advisors Liao, J. (ThermoFisher, Carlsbad, CA, USA, Personal Communication 2025), which utilized 200 µL of clarified mosquito homogenate, with extracted RNA eluted into 90 µL of elution buffer. Appendix B details the three RNA extraction protocols used during the study period.

### 2.6. RAMP^®^ WNV Testing

RAMP^®^ WNV, an immunochromatographic antigen-detection assay, was performed according to the manufacturer-provided protocol (Response Biomedical Corporation, Burnaby, BC, Canada). Pools resulting in less than 300 RAMP^®^ units were considered an equivocal result and were sent to the Idaho Bureau of Laboratories (IBL) for confirmatory testing via RT-qPCR. RAMP^®^ values of 300 and greater were considered a WNV-positive result and were not submitted for confirmatory testing [13,22]. Results of confirmatory tests were typically available within 7–10 business days, but up to 14 days during peak mosquito season.

### 2.7. CoDx North America West Triplex WNV, WEEV, and SLEV RT-qPCR Testing

During 2023 and early 2024, CoDx North American West (CoDx NAM-w) RT-qPCR kits were used while developing the molecular laboratory space and protocols (Co-Diagnostics Inc., Salt Lake City, UT, USA). RT-qPCR was performed per the manufacturer’s instructions, with pools considered positive for cycle threshold (CT) values below 40 and both positive and negative controls passing. A total of 310 pools during this period were tested in duplicate by IBL, with an aliquot of each pool submitted to validate the CoDx NAM-w results.

### 2.8. Triplex RT-qPCR Detection of WNV, WEEV, and SLEV

Triplex RT-qPCR reactions followed the protocol established in Brault, Fang, and Reisen 2015 [23,24,25]. Briefly, the triplex RT-qPCR used TaqMan Fast Virus (NO ROX) 1-Step Master Mix in a 20 µL reaction volume following the manufacturer’s fast cycling protocol (ThermoFisher, Applied Biosystems, Carlsbad, CA, USA). Reactions consisted of 5 µL of template RNA, 200 nmol of each probe, and 500 nmol of each primer. Dual-labeled BHQplus hydrolysis probes were used that target WNV, WEEV, and SLEV with probe assignments: FAM: WNV, CIV-550: WEEV, and Quasar^®^ 670: SLEV. Probes and primers for each virus species were ordered from Biosearch Technologies (Petaluma, CA, USA). Details of probe and primer formulations, concentrations, and reaction conditions are available in Appendix A. Reactions were run in 0.1 mL 4-strip tubes on a Rotor-Gene Q 5 Channel thermocycler (Qiagen, Germantown, MD, USA) with the following cycling profile: reverse transcription at 50 °C for 10 min, activation at 95 °C for 50 s, and 40 cycles of 95 °C for 15 s and 60 °C for 30 s. Each run included positive controls consisting of 5 µL extracted RNA template for each virus mixed in a 1:1:1 ratio, and a 5 µL negative control of distilled water. Positive control template RNA was prepared from stock virus provided by the CDC Arbovirus Reference Collection and extracted via the centrifugation protocol referenced in Section 2.5. Pools were considered positive for CT values below 37 with both positive and no-template controls (NTC) passing.

### 2.9. Spatial Data Harmonization and Biological Site Assignment

To address inconsistencies in historical trap naming and small-scale annual relocations, all trap coordinates from 2021 to 2025 were consolidated into a master dataset. Trap sites frequently need to be adjusted to accommodate new construction, the removal of trees and farmland, new homeowners, etc. Sites with latitude and longitude coordinates within 600 m were grouped into unique biological sites representing biologically coherent sampling areas and operationally consistent locations [26]. Distances were calculated using haversine metrics. Name variants and related metadata were archived in a NameHistory field. Each mosquito pool inherited its biological site ID based on coordinate matching, enabling consistent multi-year spatial analyses. The process of spatial assignment of biological site ID can be found in Appendix D.

### 2.10. Data Integration and Geographic Analysis

Mosquito identification, pooling, testing results, and spatial assignment data were merged into a unified dataset. Annual and cumulative WNV-positive pools (2021–2025) were aggregated at both individual trap locations and grouped biological site IDs. Spatial analysis was conducted using QGIS Desktop version 3.44.7 Solothurn (QGIS Development Team, Beaverton, OR, USA) to generate geospatial maps displaying WNV-positive pools, as well as negative pools with marker color indicating testing results and marker size scaled to the total number of pools collected. These visualizations were used to identify areas with higher sampling intensity and increased WNV activity.

## 3. Results

**Figure 1:**
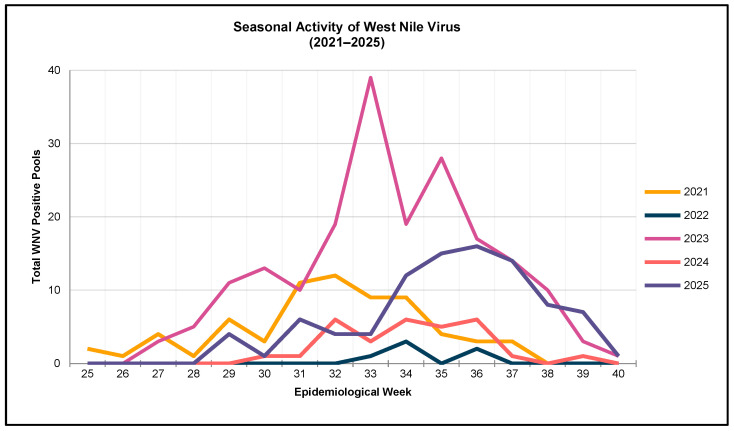
Seasonal activity of West Nile virus from mosquito pools testing positive for WNV for the years 2021 through 2025.

Across the five-year surveillance period, CCMAD process improvements allowed for increasing numbers of mosquito pools to be tested each year as laboratory capacity expanded and trapping coverage broadened. WNV-positive pools were detected in every year of the dataset, with seasonal peaks reliably occurring from late July through early September, consistent with regional patterns observed across the western U.S. [7,27] (Figure 1). Although peak virus activity varied across years, WNV activity typically peaks in epidemiological weeks 31 through 37. 2021, 2023, and 2025 were the highest years of viral activity, as indicated by WNV-positive pools during the five-year reporting period, with a peak activity in weeks 32, 33, and 36 (Figure 1), respectively. In 2025, CCMAD saw the greatest deviation in peak activity, with week 36 showing the most activity (Figure 1). Visualizing historical trendlines in the surveillance data (Figure 1) proves useful for resource allocation. In 2023 and 2025, the highest WNV activity was detected, with 192 and 92 positive pools detected each year, respectively (Table 1). From 2021 to 2025, there were no detections of WEEV or SLEV in pools tested via RT-qPCR by IBL or in-house testing since 2023.

**Figure 2:**
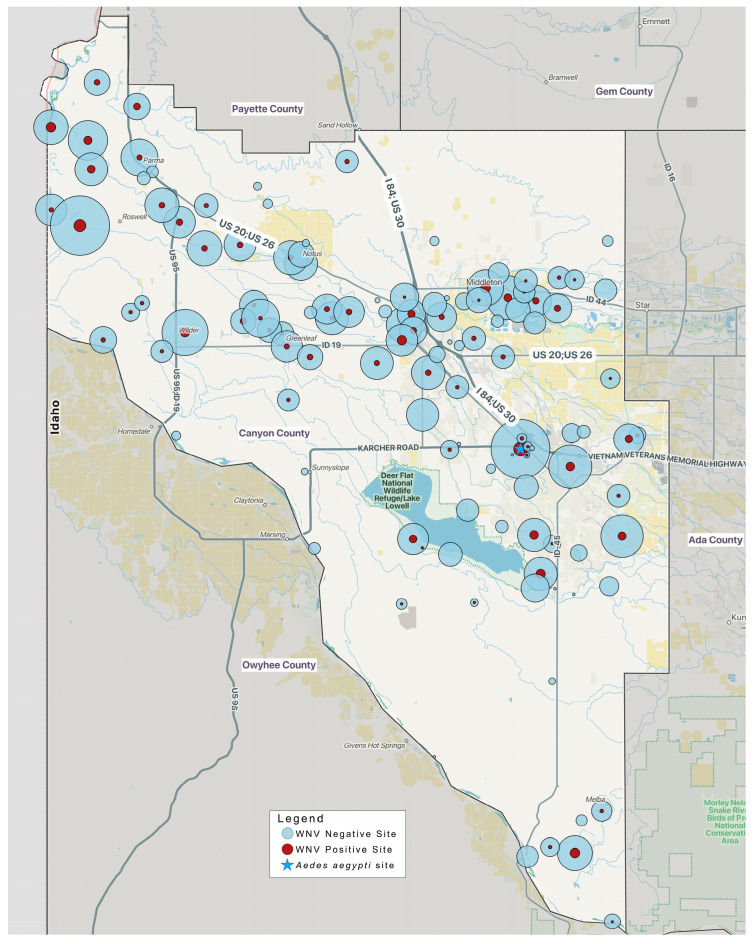
Map of Canyon County, Idaho, with the total WNV-positive and negative *Culex* spp. pools by location for the years 2021–2025. Red circles indicate pools testing positive for WNV, while blue represents pools testing negative during the five-year period. Circle size increases relative to the total number of pools from that site resulting in positive or negative results from 2021 through 2025. The location for the detection of one *Aedes aegypti* specimen is indicated by a blue star. Basemap data © OpenStreetMap contributors; OpenStreetMap data are available under the Open Database License (ODbL). Derivative data © NextGIS Data (data.nextgis.com), also available under ODbL. Map styling and design © NextGIS (CC BY 4.0). QGIS symbols © Yury Ryabov and Pavel Sergeev (CC BY 3.0).

While annual magnitudes varied, several areas within the County demonstrated visual patterns suggesting elevated activity, although not statistically tested. Biological site aggregation revealed relatively stable, recurrent multi-year transmission zones in the northern portions of the County, which consists of the I-84 and Highway 20/26 corridors, that were not apparent when examining raw trap-level data (Figure 2). These zones included cemetery-adjacent corridors, irrigated agricultural edges, and riparian interfaces known to support high *Culex* productivity and confirm the rationale for targeting these areas for the surveillance and control of vectors and arboviruses [26,28]. As illustrated in Figure 2, surveillance sites occur County-wide; however, the I-84 corridor, which includes the Boise River basin, as seen in the northeast portions of the County, stands out as a region with repeated viral isolations and a high trap density. This area of the County is both urban (along the I-84 corridor) and contains floodplains from the Boise River, especially in the northwestern portions of the County along the Highway 20/26 corridor (as seen in the northwest portions of the County). These two habitat zones create abundant *Cx. pipiens* and *Cx. tarsalis* populations, respectively.

Aggregating the sites and geographically visualizing them allowed CCMAD staff to better identify redundant collection sites and previously unseen gaps in our surveillance network. As a result, the number of trap sites has been reduced from a high of 127 sites in 2022 to 100 in 2025. Despite this reduction in the number of traps, the number of collected *Culex* mosquitoes and pools tested increased each year (Table 1).

**Figure 3:**
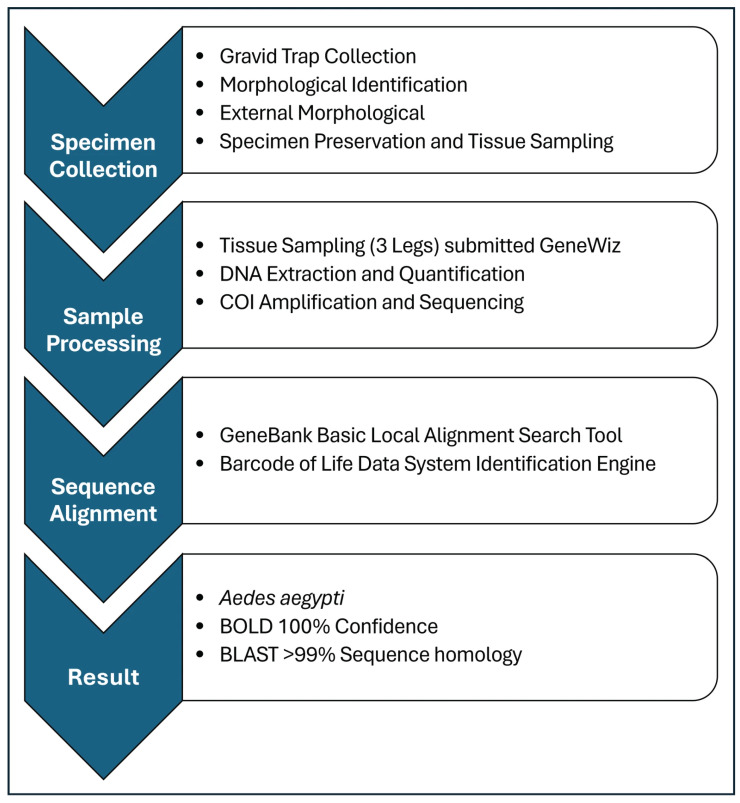
Workflow process to identify the single *Ae. aegypti* collected in Canyon County through DNA barcoding of the mitochondrial COI segment.

In 2025, a single adult female *Ae. aegypti* was collected during routine gravid trapping in a trap placed outdoors near a shopping mall. Identification was initially performed via morphological examination and was validated by external experts. Due to the specimen being heavily damaged, identification was further confirmed through barcode sequencing of the cytochrome oxidase subunit 1 (COI) sequence [29,30]. The sequence showed a greater than 99% identity to reference *Ae. aegypti* sequences in the Core nucleotide database (core_nt) using the Megablast algorithm in the Basic Alignment Search Tool (nucleotide) (BLASTN) (NCBI, Bethesda, MD, USA) and a 100% confidence as *Ae. aegypti* in the Barcode of Life Data System (BOLD) (BOLD Systems, Guelph, Ontario, Canada) identification system search [31,32,33,34]. One locally collected *Ochlerotatus dorsalis* and one pinned *Ae. aegypti* collected in 2020 from St. Augustine, FL, were included as positive controls for DNA extraction, COI segment amplification, sequencing of COI products, and sequence alignment through BLASTN and BOLD systems. The specimen’s processing and confirmation workflow is illustrated in Figure 3.

After the identification of the specimen on 11 August 2025, surveillance was enhanced in a 1.5 km (1 mile) radius around the initial trap site. Subsequent targeted trapping occurred from 15 August 2025 through 30 September 2025 and relied on weekly collections from ovicups (*n* = 24), BG-Sentinel 2 traps (*n* = 36), gravid traps (*n* = 6), and EVS traps (*n* = 18), with all traps placed outdoors. These collections did not yield additional *Ae. aegypti* specimens, suggesting either a transient introduction or a very small, localized presence consistent with patterns observed during early establishment in other temperate regions [35].

The implementation of in-house triplex RT-qPCR and automated extraction doubled CCMAD’s annual pool-processing capacity (Table 1). Through the adoption of various testing assays from year to year, from RAMP^®^ assays to in-house RT-qPCR, the number of pools tested has increased each year since the implementation of our modernization plan. Another distinct benefit of increasing laboratory capacity has been the reduced per-sample test and consumable costs compared with immunoassay-based RAMP^®^ testing. Prior evaluations of automated nucleic acid extraction paired with multiplex RT-qPCR in arbovirus surveillance laboratories have reported improved laboratory efficiency and scalability, supporting the similar operational benefits observed here [36]. As expected from other laboratories [1,6,36], CCMAD has also seen a reduced per-pool cost with each optimization of its testing platform. While the RAMP^®^ assay was used, the per-pool test cost was $21. The Co-Dx platform in CCMAD’s configuration averaged $14 per pool tested. Moving beyond the preformulated Co-Dx NAM-w kits to the adapted Brault PCR protocol, along with automated RNA extraction, has allowed our costs to decrease to $10 per pool. It should be noted, of course, that there is the considerable expense of the staff, facilities, and equipment required to move beyond the basic RAMP^®^ assay or Co-Dx NAM-w testing protocols.

## 4. Discussion

In response to regional arboviral presence, CCMAD initiated a multi-year process to expand their laboratory capacity. The expansion of the District’s surveillance and diagnostic capacity is critical for strengthening the early detection of arboviruses in the region the District serves, further developing rapid response to vector-borne disease threats within southwest Idaho. It was evident from previous infections that arboviruses such as WNV, SLEV, and WEEV could circulate silently in mosquito and host populations before human outbreaks occur [1,7]. The development of automated RNA extraction procedures coupled with the utilization of a validated triplex RT-qPCR assay has substantially increased CCMAD’s diagnostic throughput and analytical ability to detect WNV, WEEV, and SLEV in mosquito samples prior to human cases occurring (Table 1). These practices are consistent with national guidance emphasizing mosquito-based surveillance as the primary early warning system for arboviral transmission and operational decision-making [8]. These improvements align with national recommendations from the CDC and other national agencies encouraging local vector programs to establish sustainable, in-house molecular diagnostic capacity [11,12] that supports integrated mosquito management programs in protecting public health and reducing the burden of both vectoring and pestiferous mosquito populations for their constituents.

Without in-house molecular diagnostic tools, such as real-time PCR, many local programs are forced to rely on external labs, which can delay detection and response protocols. By investing in laboratory infrastructure at the district level, mosquito control agencies can conduct more frequent and timely testing of mosquito pools, thus enabling the rapid identification of viral activity and targeted vector control interventions before human transmission begins. Strengthening the laboratory capacity also ensures the early identification of infected vectors and human cases, which is critical for activating targeted mosquito control and public health messaging [1]. Visualizing trend lines (Figure 1) provides district management with a greater resolution of peak mosquito and virus activity, allowing for predictive management strategies and improved chemical, budget, and staff forecasting.

The need for local and regional laboratory infrastructure is emphasized due to the many limitations of relying on clinical diagnosis for arboviral diseases. For example, Zika virus infections can mimic other febrile illnesses, making laboratory confirmation via molecular methods essential for outbreak detection and response [37]. Although these authors referred to the detection of Zika virus within humans, Petersen et al. [1] highlight that laboratory surveillance is central to managing WNV epidemics. Delays in detecting the virus in mosquito vectors can often result in unmitigated spread. California’s long-term experience with WNV illustrates the value of integrated laboratory and field surveillance. From 2003 to 2018, a coordinated system involving local mosquito control districts and in-house diagnostic capacity enabled the real-time monitoring of viral activity in mosquito populations and dead birds, which closely predicted human WNV cases [7].

Beyond early detection, enhanced laboratory capabilities also improve the understanding of co-infection dynamics and inform outbreak management strategies. In Brazil, da Conceição et al. [38] demonstrated that molecular testing uncovered previously unrecognized co-infections of DENV-2 and Zika virus, which would have been missed using traditional serologic methods. Studies have also called for the integration of laboratory diagnostics into broader dengue surveillance systems across the Asia-Pacific, asserting that lab-informed responses reduce the scope and severity of outbreaks there and in other areas [39,40]. Though these studies are in human epidemiology and public health, they parallel the efforts of mosquito abatement programs tasked with protecting public health from mosquito-borne disease. In this manner, expanding our laboratory’s capacity and summarizing the data in real-time reports and graphs is an essential element of the District’s long-term mission to build a responsive, proactive program that can manage the increasing burden of arboviral diseases in the region and ensure that timely mosquito abatement is conducted efficiently to reduce the risk of human transmission.

In addition to providing for the early and accurate identification of mosquito-borne arboviruses, CCMAD’s capacity-building has added staff to provide expertise and consistency in data collection and recording. The multi-year analysis and site aggregation efforts performed in 2025 indicated that WNV transmission in Canyon County is shaped by persistent ecological features rather than isolated introductions (Figure 2). Furthermore, biological site aggregation revealed clusters of activity centered around irrigated agricultural systems, riparian corridors, and cemetery-associated vegetation. Although it is not surprising that these landscapes support *Culex* breeding and high vector–host contact rates [26,28], as evidenced by the high number of infected mosquitoes collected in these trap locations, visually delineating these areas allows for the geographic prioritization of management operations. Interestingly, these zones also remain active across multiple years, underscoring their importance for operational prioritization.

Historically, the Deer Flat National Wildlife Refuge (DFNWR) around Lake Lowell located in the center of Canyon County has been a heavy production zone for pestiferous *Aedes*, *Anopheles*, and *Culiseta* species due to the seasonal snowmelt flooding and preferential freshwater habitat associated with these species. The District’s surveillance efforts matched this burdensome concern from residents. However, as seen in Figure 2, the number of WNV-positives in this area is relatively low in comparison to other areas within the County, most likely because the primary WNV vectors in Idaho prefer more disturbed habitats. Visualizing the data in this fashion does not provide statistically significant hotspots but has allowed our staff to reduce the number of traps in the Deer Flat NWR area while maintaining the needed resolution to detect WNV-carrying mosquitoes and respond to *Aedes* hatch-offs in a timely manner based on larval surveillance and service requests from County residents.

Furthermore, harmonizing site histories resolved inconsistencies arising from staff turnover, varying naming conventions, and minor relocations. This spatial standardization improved the interpretability of multi-year patterns and reduced the noise in cumulative mapping. Cumulative WNV-positive biological sites (Figure 2) showed persistent hotspots with repeated WNV activity across multiple years; spatial stability despite interannual variation; and areas of operational significance where increased surveillance or mosquito management operations may yield the greatest impact. Standardizing historical trap locations into biological sites also allowed CCMAD to gain the ability to track meaningful changes in transmission patterns independent of shifting trap names or minor spatial adjustments. This spatial harmonization supports the more precise deployment of adulticide efforts, refines trap allocation, and targets follow-up in areas with sustained, elevated risk.

The significance of having staff trained in entomologic identifications cannot be understated. This in-house capacity allows for the identification of new, invasive mosquito species. In 2025, CCMAD reported the first confirmed detection of *Ae. aegypti* in Idaho (manuscript in preparation). This finding highlights the importance of maintaining a surveillance system capable of detecting invasive vectors whose ranges are expanding due to climate, trade, and human movement patterns [14,15,35]. It is well known that the delayed detection of *Ae. aegypti* has been linked to explosive outbreaks, such as in the southern U.S. states and Latin America, where vector populations expanded before public health systems and mosquito abatement programs could respond [14,41,42,43]. Although intensive sampling did not identify additional *Ae. aegypti* specimens, it is likely that rapid morphological and molecular confirmation informed a timely and targeted eradication effort, potentially preventing the establishment of *Ae. aegypti* in Canyon County, Idaho. Furthermore, the collection of this morphologically damaged specimen illuminated the need to create a reference DNA barcode database for Idaho mosquitoes to confirm identifications of endemic and newly introduced species.

Overall, sample-processing times have been streamlined from 2022 through 2025, allowing for reduced turnaround times. In 2025, testing results were reduced to same-day results delivered to operational teams prior to planning adulticide operations for that night and larvicide operations the next day. Same day arbovirus testing results enabled rapid operational adjustments during periods of elevated arbovirus transmission. Despite 2025 being a year of higher WNV activity, we had no reported human cases within Canyon County, which in part is a result of the increased arbovirus testing capacity and decreased reporting times of positive detections used to inform mosquito abatement operations.

Given the overlapping strategy of our laboratory expansion, it is difficult to conclude that one assay type has distinct time saving advantages. From 2020 to 2022, CCMAD relied exclusively on the RAMP^®^ immunochromatographic antigen-detection assay, with confirmation analysis performed at the Idaho Bureau of Laboratories (IBL); however, by late 2022, throughput was already increasing due to recently hired, dedicated staff and improved pool-processing equipment, e.g., centrifuges and the bead-mill homogenizer. In 2023, the District adopted triplex RT-qPCR with Co-Dx NAM-w kits to detect WNV, WEEV, and SLEV. Initially, triplex RT-qPCR testing overlapped with the RAMP^®^ tests in using non-expired stock. During the optimization of the Co-Dx protocol, samples were tested in duplicate by CCMAD and IBL to confirm the results of the Co-Dx NAM-w triplex kit and to allow for confirmation testing with the NAM-W triplex PCR kit. The initial evaluation of the CoDx NAM-w yielded false positives for SLEV and false negatives for WNV when the results were verified by the IBL. To increase the confidence in RT-qPCR results, a previously published protocol [23] was adopted and validated in 2024. Once validated and optimized, the results aligned with confirmatory testing, and the protocol is the current standard used by CCMAD.

In 2025, the addition of a QIAvac 24 vacuum manifold greatly decreased RNA extraction times. The improved extraction times coupled with the protocol adopted from Brault et al. [23] provided streamlined results and increased confidence in our testing platform. These improvements, along with past confirmatory work, allowed fewer samples to be sent to IBL, decreasing the time to report WNV detections to operational staff. Decreased reporting times allowed operational staff to respond to increasing populations or virus presence within the same day for adulticide operations and the next day for larvicide teams. With these capacity improvements, justification to purchase a Kingfisher Flex automated nucleic acid extraction system was available, and the instrument was procured in August 2025. With the addition of an automated nucleic acid processor, sample preparation is expected to experience a significant increase in efficiency in 2026, further supporting our operational teams.

The expanded laboratory capacity from 2022 to 2025 has allowed greater flexibility and improved workflows, enabling the collection and identification of introduced species with improved accuracy, which allows for improved and timely intervention strategies by operational staff. These intervention strategies range from increased public notifications (e.g., press releases), additional surveillance operations (larval and adult), to enhanced larvicide applications via backpack equipment and drone operators. In addition, nightly ULV truck-mounted adulticide operations are often deployed. In the past, additional surveillance sites would be logged and recorded as new site locations, creating temporary sites and incongruent site data from year to year. With the newly aggregated biological site protocols, enhanced surveillance traps near arboviral detections or invasive species retain the original site designations and allow for improved data integrity within sites.

The combination of enhanced laboratory capacity, harmonized spatial datasets, and refined operational workflows allowed CCMAD to improve both the sensitivity and the responsiveness of its arbovirus surveillance system. These integrated upgrades strengthen the District’s ability to detect spatial shifts in WNV activity and identify emerging vector threats. Continued support for laboratory infrastructure, data integration, and workforce training will allow CCMAD to sustain and enhance its surveillance capacity into the future as environmental conditions and vector populations evolve.

## 5. Conclusions

This study demonstrates that targeted investments in local laboratory infrastructure, standardized data management, and workforce expertise can substantially strengthen arbovirus surveillance capacity at the district level. By implementing validated in-house triplex RT-qPCR testing and integrating automated RNA extraction, the Canyon County Mosquito Abatement District increased diagnostic throughput and reduced reporting delays while maintaining consistency across multiple surveillance seasons. These improvements enabled a more timely interpretation of mosquito infection data during periods of elevated West Nile virus activity.

The harmonization of historical trap locations into biologically defined sampling units further demonstrates the value of spatial standardization for multi-year arbovirus analyses. Grouping traps based on geographic proximity rather than variable site naming revealed persistent areas of WNV activity associated with stable ecological features, supporting a more consistent interpretation of temporal trends and improved operational planning. This approach illustrates a practical method for resolving common challenges in legacy surveillance datasets and facilitates longitudinal comparisons without a reliance on static trap identifiers.

The enhanced surveillance framework, coupled with trained staff, also enabled the first confirmed detection of *Aedes aegypti* in Idaho. Morphological identification followed by molecular confirmation underscores the importance of maintaining local taxonomic and molecular capacity for the early detection of invasive vectors. Such capacity is essential for rapid follow-up surveillance and risk assessment in regions where vector distributions are actively changing.

## Figures and Tables

**Table 1:** Total *Culex*, *Culex pipiens*, *Culex tarsalis*, and *Culex erythrothorax* mosquitoes, pools tested and result totals.

	Year	Mosquitoes	Pools	WNV-Positive	Negative
Total *Culex*	2021	39,178	1313	68	1245
	2022	34,381	1580	6	1574
	2023	38,767	2599	192	2407
	2024	57,174	2697	30	2667
	2025	69,413	3292	92	3200
*Culex pipiens*	2021	22,598	754	45	709
	2022	20,537	897	4	893
	2023	21,469	1391	118	1273
	2024	30,831	1440	14	1426
	2025	41,930	1920	62	1858
*Culex tarsalis*	2021	15,101	504	22	482
	2022	11,929	580	2	578
	2023	16,012	1128	69	1059
	2024	21,490	1140	15	1125
	2025	23,118	1242	30	1212
*Culex erythrothorax*	2021	1479	55	1	54
	2022	1915	103	0	103
	2023	1286	80	5	75
	2024	4853	117	1	116
	2025	4365	130	0	130

WNV-positive and negative pools for total *Culex*, *Culex pipiens*, *Culex tarsalis*, and *Culex erythrothorax*, aggregated by year for mosquitoes collected and tested within Canyon County from 2021 through 2025.

## Data Availability

Data can be requested from the corresponding author or the Canyon County Mosquito Abatement District.

## Abbreviations

The following abbreviations are used in this manuscript:

Abbreviation	Definition
*Ae*.	*Aedes*
BG-Sentinel 2	BG-Sentinel 2 mosquito trap
BOLD	Barcode of Life Data System
BLASTN	Basic Alignment Search Tool (nucleotide)
CDC	Centers for Disease Control and Prevention
COI	Cytochrome c oxidase subunit I (mitochondrial barcode region)
CT	Cycle threshold
*Cx*.	*Culex*
CCMAD	Canyon County Mosquito Abatement District
DENV	Dengue virus
DNA	Deoxyribonucleic acid
EBO	Empty but operational (trap status code)
EVS	Encephalitis virus surveillance trap
EPA	Environmental Protection Agency
GPS	Global Positioning System (SS2.1; Appendix C.3)
IBL	Idaho Bureau of Laboratories
IDHW	Idaho Department of Health and Welfare
NAM-w	CoDx North America West RT-qPCR kit
NACCHO	National Association of County and City Health Officials
NCBI	National Center for Biotechnology Information
NTC	No-template control
PCR	Polymerase chain reaction
PFU	Plaque-forming units
qPCR	Quantitative polymerase chain reaction
RT-qPCR	Reverse-transcription quantitative polymerase chain reaction
QIAvac	Qiagen vacuum manifold extraction system
RNA	Ribonucleic acid
RAMP^®^	Rapid Analyte Measurement Platform assay
SLEV	St. Louis encephalitis virus
TaqMan	Hydrolysis-probe quantitative PCR chemistry
ULV	Ultra-low volume (pg. 13)
WEEV	Western equine encephalitis virus
WNV	West Nile virus

## Appendix A. Triplex RT-qPCR Protocol

### Appendix A.1. Primer and Probe Sequences

Primer and probe sequences for WNV, WEEV, and SLEV were sourced from published assays (Lanciotti and Kerst 2001 [44]; Brault et al. 2015 [23]; Lanciotti et al. 2000 [24]; Shi et al. 2001 [25]). Final reaction concentrations: 500 nM primers, 200 nM probes.

**Target**	**Oligo**	**Sequence (5′→3′)**	**Label**
SLEV [44]	Forward	CTGGCTGTCGGAGGGATTCT	
	Reverse	TAGGTCAATTGCACATCCCG	
	Probe	TCTGGCGACCAGCGTGCAAGCCG	Quasar 670/BHQ-2
WEEV [23]	Forward	GTCTTCAACTCGCCGGATCTTA	
	Reverse	GGTGTCAAGCGGAATGGAA	
	Probe	CACACAGACCACTCAGTGCAAGGTAAACTGC	CIV-550/BHQ-1
WNV [24,25]	Forward	TCAGCGATCTCTCCACCAAAG	
	Reverse	GGGTCAGCACGTTTGTCATTG	
	Probe	TGCCCGACCATGGGAGAAGCTC	FAM/BHQ-1

### Appendix A.2. Reaction Mixture (20 µL Total Volume)

2.0 µL 10× assay mix,

7.0 µL nuclease-free water,

5.0 µL TaqMan Fast Virus 1-Step Master Mix (No ROX),

6.0 µL RNA template.

### Appendix A.3. Thermal Cycling Conditions

Reverse Transcription: 50 °C for 10 min (one cycle);

Activation: 95 °C for 50 s (one cycle);

Denaturation: 95 °C for 15 s (40 cycles);

Annealing/Extension: 60 °C for 30 s, with fluorescence acquisition (40 cycles);

Total Reaction Volume: 20 µL.

### Appendix A.4. Viral Controls and Expected CT

**Stock Virus**	**PFU/mL**	**Dilution**	**Expected CT**
SLEV FTA Card	150,000	1:160	~31
WEEV FTA Card	12,500,000	1:80	~30
WNV Lysate	1,000,000	1:100	~25

## Appendix B. RNA Extraction Protocols

### Appendix B.1. QIAamp Viral RNA Mini Kit (Centrifuge Method)

1. Input: 140 µL of clarified mosquito homogenate.
2. Add 560 µL of buffer AVL and incubate at room temperature for 10 min.
3. Add 560 µL of ethanol and mix thoroughly.
4. Load lysate onto spin column in 600–700 µL aliquots; centrifuge 6000× *g* for 1 min.
5. Wash column with 500 µL of buffer AW1; centrifuge 6000× *g* for 1 min.
6. Wash column with 500 µL of buffer AW2; centrifuge 20,000× *g* for 3 min.
7. Elute RNA with 50 µL of buffer AVE; centrifuge 6000× *g* for 1 min.
8. Output: 50 µL RNA (stored at −80 °C).

### Appendix B.2. QIAamp Viral RNA Mini Kit (QIAvac Vacuum Manifold Method)

1. Input: 140 µL of clarified homogenate.
2. Combine sample with 560 µL of buffer AVL; incubate 10 min.
3. Add 560 µL of ethanol and mix.
4. Load onto QIAvac column and apply vacuum to draw entire lysate through.
5. Wash with 500 µL of buffer AW1 under vacuum.
6. Wash with 500 µL of buffer AW2 under vacuum.
7. Remove column and dry-spin 3 min at 20,000× *g*.
8. Elute RNA with 50 µL of buffer AVE; centrifuge 6000× *g* for 1 min.

### Appendix B.3. MagMAX CORE Extraction Using the KingFisher Flex

Homogenization (Pre-Extraction)

1. Add mosquito pool and copper BB to tube with 800 µL of medium.
2. Homogenize 2 × 20 s at 6000 rpm with 5 s pause.
3. Clarify at 13,000 rpm for 3 min.

KingFisher workflow (ThermoFisher CORE protocol)

4. Combine clarified lysate with Proteinase K and Lysis/Binding Solution with magnetic beads.
5. Prepare two wash plates (500 µL wash 1, 500 µL wash 2) and one elution plate (90 µL elution buffer).
6. Load plates and tip comb; run MagMAX CORE program.
7. Output: ~90 µL RNA.

## Appendix C. Data Storage Structure, Contents, and Internal Relationships

This appendix describes how mosquito surveillance data are generated, stored, and related to one another within CCMAD’s Excel-based system. The workflow consists of three distinct datasets, Location Data, Identification Data, and Pool Data, each created sequentially as mosquitoes move from field collection through laboratory testing. Although stored in separate Excel files, the datasets remain linked through consistent identifiers and shared metadata.

### Appendix C.1. Overview of Data Generation and Flow

Mosquito surveillance data are created in a fixed order:

1. Trap setting and collection,
2. Trap identification and pool creation,
3. Laboratory testing and pool-level results.

Each stage produces a dataset that carries forward the information needed to track mosquitoes from field collection through final arbovirus detection.

### Appendix C.2. Dataset 1: Location Data (Site Reference File)

The Location Data file stores static information about every trap site used by the District. This file does not change day-to-day but is referenced continually by staff.

Contents:

• SiteID (district-defined site code);
• SiteName (if applicable);
• Latitude, Longitude (GPS coordinates);
• Location type (park, agricultural field, cemetery, residential, etc., optional);
• Notes (access constraints, property changes, etc.).

Purpose:

• This provides the geographic reference needed to link trap-level data to spatial analyses.
• Relationships.
• Identification Data link to Location Data via SiteID.

Pool Data inherits geographic information indirectly through Identification Data.

### Appendix C.3. Dataset 2: Identification Data (Trap-Level Mosquito Identification)

This dataset is created after traps are retrieved from the field.

Data Generation:

1. Each trap is labeled in the field with a SiteID and collection date.
2. After collection, mosquitoes are euthanized and identified to species.
3. Counts for each species are recorded.

Contents:

• CollectionDate,
• SiteID,
• TrapType (EVS, gravid, BG-Sentinel),
• SpeciesIdentified (*Cx. tarsalis*, *Cx. pipiens*, etc.),
• NumberOfIndividuals for each species,
• TrapStatus (malfunction, not set, empty but operational [EBO]),
• Field or Lab Technician,
• Notes (weather, trap interference, unusual catches).

Purpose:

This provides the biological inventory for each trap and determines how pools are formed.

Relationships:

• Pools are created directly from this data.
• Pool Data reference Identification Data through:
• SiteID,
• CollectionDate,
• Species,
• FemalesPerPool.

Identification Data therefore acts as the parent record for all pools.

### Appendix C.4. Dataset 3: Pool Data (Pool Metadata + Test Results)

*Culex* mosquitoes are pooled after identification. Each pool contains mosquitoes:

• From a single species,
• From one SiteID,
• From one CollectionDate,
• With 1–50 individuals.

If more than 50 mosquitoes of the same species are collected at a site on a given date, additional pools are created.

Pool ID Assignment

Pools receive a unique identifier:

YY-####.

• YY = two-digit year.
• #### = sequential pool number assigned in the order pools are created.

Example: 25-1010 = 1010th pool created in 2025.

Contents of Pool Data:

• PoolID (YY-####),
• CollectionDate,
• SiteID,
• Species,
• FemalesPerPool,
• HomogenizationDate,
• ExtractionMethod (spin, vacuum, KingFisher),
• TestingBatch (identifier for a set of samples processed in parallel),
• CT_WNV, CT_WEEV, CT_SLEV,
• Result (Positive, Negative, Equivocal),
• Notes (difficult homogenization, retests, etc.).

Purpose

This records all metadata and results for each pool tested for arboviruses.

Relationships

Pool Data can be linked back to identification and location datasets through the following:

SiteID → connects to Location Data;

Species, CollectionDate, FemalesPerPool → connects to Identification Data;

PoolID → connects to laboratory testing and operational response logs.

Thus, even though the datasets exist as separate Excel sheets, their relationships remain consistent and traceable.

### Appendix C.5. Summary of Relationships Between the Three Datasets

**Dataset**	**Contains**	**Key Linking Fields**	**Relates to**
Location Data	Site coordinates, static reference info	SiteID	Identification Data, Pool Data (Indirectly)
Identification Data	Species counts per trap	SiteID, CollectionDate, Species	Pool Data
Pool Data	Pools + test results	PoolID, SiteID, CollectionDate, Species	Identification Data, Laboratory Testing

Logical flow of data:

Location → Identification → Pool → Laboratory Results.

This structure ensures that every mosquito recorded in the system can be traced from its collection site through identification, pooling, extraction, and final RT-qPCR results.

## Appendix D. Spatial Assignment and Biological Sites

Performed in Microsoft Excel™

Procedure:

1. Combine trap coordinates.
2. Compute haversine distances.
3. Group points within 600 m.
4. Assign biological site ID.
5. Record NameHistory.

Output:

Harmonized multi-year spatial dataset.
